# Effect of NZ2114 against *Streptococcus dysgalactiae* biofilms and its application in murine mastitis model

**DOI:** 10.3389/fmicb.2022.1010148

**Published:** 2022-09-15

**Authors:** Na Yang, Qingjuan Zhang, Ruoyu Mao, Ya Hao, Xuanxuan Ma, Da Teng, Huan Fan, Jianhua Wang

**Affiliations:** ^1^Team of AMP & Alternatives to Antibiotics, Gene Engineering Laboratory, Feed Research Institute, Chinese Academy of Agricultural Sciences, Beijing, China; ^2^Key Laboratory of Feed Biotechnology, Ministry of Agriculture and Rural Affairs, Beijing, China; ^3^Tianjin Animal Science and Veterinary Research Institute, Tianjin, China; ^4^College of Life Sciences, Tianjin Normal University, Tianjin, China

**Keywords:** *Streptococcus dysgalactiae*, antimicrobial peptide, NZ2114, bovine mastitis, biofilms

## Abstract

Bovine mastitis caused by *Streptococcus dysgalactiae* (*S. dysgalactiae*) is usually treated with antibiotics, which may potentially increase drug resistance as the abuse. NZ2114, a variant of fungal defensin plectasin, displayed a potent antibacterial activity against *S. dysgalactiae*. The inhibition/eradication effect of the antimicrobial peptide NZ2114 on the early/mature biofilm of *S. dysgalactiae* CVCC 3938 was evaluated, as well as the elimination of bacteria in mature biofilms. In this study, NZ2114 displayed potent antibacterial activity against *S. dysgalactiae* CVCC 3938 and three clinical isolated *S. dysgalactiae* strains (0.11-0.45 μM). The early biofilm inhibition of *S. dysgalactiae* CVCC 3938 was 55.5–85.9% after treatment with NZ2114 at concentrations of 1–16 × MIC, which was better than that of vancomycin at the same concentration. The mature biofilm eradication rate was up to 92.7–97.6% with the increasing concentration (2–16 × MIC) of NZ2114, and the eradication rate did not change significantly with further increase of NZ2114 concentration, while the biofilm eradication rate of vancomycin-treated group at the same concentration remained at 92.5%. NZ2114 reduced the number of persister bacteria in biofilm. Scanning electron microscopy (SEM) and confocal laser scanning microscopy (CLSM) further demonstrated that NZ2114 could effectively reduce the biofilm thickness and bacterial number of *S. dysgalactiae* CVCC 3938. *In vivo* therapeutic effect of NZ2114 on murine mastitis model showed that NZ2114 was better than vancomycin in alleviating mammary gland inflammation by regulating cytokines production, inhibiting bacterial proliferation, and reducing the number of mammary gland bacteria. These data suggested that NZ2114 is a potential peptide candidate for the treatment of mastitis.

## Introduction

Bovine mastitis is an inflammatory disease caused by variety of pathogenic microorganisms with invasion of mammary gland tissue and long-term infection. The disease reduces milk production, augments breast necrosis rate, and even leads to death of dairy cows, which seriously threatens the health of dairy cows and affects the development of the dairy industry (Melchior et al., 2006). *Streptococcus dysgalactiae* (*S. dysgalactiae*) is one of the major *Streptococcus* species that cause mastitis (Ma et al., 2021), it is considered to be environmental pathogenic bacteria, which is parasitic in the mouth, vagina and skin of livestock, and also distributed in the environment. This pathogen can infect livestock, causing abscesses, endocarditis and mastitis. In China, the *S. dysgalactiae* is the top three strains among the main pathogenic bacteria leading to bovine mastitis, which can reach 21.35% in Jilin province (Hao et al., 2016). In addition, this bacterium also infects humans under special situations, causing bacteremia, endocarditis, meningitis, sepsis, arthritis, respiratory and skin infections and other diseases in patients (Rantala, 2014; Rantala and Tuohinen, 2014; Rutherford et al., 2014).

Biofilm is an aggregate in which microorganisms grow organically. Bacteria irreversibly adhere to the surface of inert or active entities, multiply, differentiate, and secrete some polysaccharide matrix, which wraps the bacterial community to form aggregate membrane. It had been suggested that the bacterial biofilms of *Staphylococcus aureus* (*S. aureus*), *Escherichia coli* and *Streptococcus* were highly resistant to antibiotics and host defense (Costerton et al., 1999; Olson et al., 2002; Parastan et al., 2020). Gomes et al., study showed that the formation of biofilm of *S. dysgalactiae* is an important virulence mechanism of the pathogenic causing bovine mastitis (Gomes et al., 2016). Previous studies showed that 46.7% of isolates displayed moderate or strong biofilm-forming abilities among 246 clinical *S. dysgalactiae* (Ma et al., 2017). Two biofilm formation related genes, *eno* and *napr*, were detected in 76% and 86% of *S. dysgalactiae* (*n* = 41), respectively (Kaczorek et al., 2017). The presence of biofilms in the mammary gland has led to a decrease in the effectiveness of antibiotic therapy, there are 10 to 1000 times more resistant to the effects of antibiotics, resulting in persistent infections of pathogenic bacteria, which increases the difficulty of treatment and prolongs the time of medication. In addition, some studies had reported that subinhibitory concentrations of some antibiotics could induce biofilm formation (Costa et al., 2012). Therefore, the removal of *S. dysgalactiae* biofilm has an important clinical significance.

Antimicrobial peptides (AMPs) are a class of small molecule peptides with high-efficiency antibacterial activity in innate immune system in living organism. The multifactorial antimicrobial mechanisms of AMPs derived from intracellular compatibility, high sensitivity to early warning and low resistance from multiple targets. AMPs could cover the merits of antibiotics in disease treatment and vaccines in disease prevention, and avoid their shortcomings, such as high resistance and high variation in pathogens, and high residue in animals. AMPs, antibiotics, and vaccines could complement each other, and build an iron triangle of animal health care together to halt the development of drug resistance and reduce the residues in tissues (Teng et al., 2014; Zhang et al., 2014; Hao et al., 2017, 2022; Li Z. et al., 2017; Yang et al., 2017, 2019; Wang et al., 2018; Liu et al., 2021; Zheng et al., 2021). Therefore, it has been highly expected that AMPs could exert a positive contribution to support green husbandry in terms of a new health concept One World and One Health. However, most AMPs stop at the assessment into clinical application due to their poor druggability, only a few AMPs show a promising future in drug development. It was reported that NZ2114 is the derived peptide of the first fungal defensin plectasin which targets specially Lipid II in cell wall of gram-positive bacteria, with three amino acid sites mutated (D9N, M13L, and Q14R). NZ2114 exhibited potent antimicrobial activity, with the minimum inhibitory concentration (MIC) values of 0.057–0.454 μmol/L for methicillin-resistant *S. aureus* (Chen et al., 2017). When NZ2114 was combined with hybrid catheter material, it displayed significant inhibition effect of methicillin-resistant *S. aureus* biofilms (Klein et al., 2017). NZ2114 also exhibited effective activity against *Streptococcus* (Pillar et al., 2008). In our previous study, NZ2114 had potent antimicrobial activity against *Streptococcus suis* (*S. suis*) with MIC values of 0.03–0.06 μmol/L. The *in vitro* time-kinetic results showed that NZ2114 was 99.9% bactericidal against *S. suis* CVCC 606 within 4 h at 4 × MIC concentration. In addition, the antimicrobial peptide NZ2114 had a good protective effect on mouse peritonitis infected by *S. suis*. The dose of 0.2 mg/kg NZ2114 treatment could result in a 100% survival rate of mouse, which effectively reduced the bacterial load in mice organs, regulated the release of inflammatory factors, alleviated the histopathological damage of organs and thus effectively protected mice against *S. suis* type 2 strains infection (Jiao et al., 2017). Therefore, NZ2114 is a potential antimicrobial agent to inhibit and eradicate *S. dysgalactiae* and its biofilms.

Based on the previous studies of plectasin-derived peptides in treatment of *Streptococcus* infection, this study is focus on exploring the inhibition/eradication effect of NZ2114 on *S. dysgalactiae* biofilm *in vitro* and the therapeutic effect of mouse mastitis *in vivo*.

## Materials and methods

### Strains, mice and reagents

*Streptococcus dysgalactiae* CVCC 3938 was purchased from the China Veterinary Culture Collection Center (CVCC) (Beijing, China). The tested strains of *S. dysgalactiae* CAU-FRI 1-3 isolated from bovine mastitis were obtained from China Agricultural University. The four-day post-parturient SPF ICR female mice were purchased from Vital River Laboratories (Beijing, China), kept in a sterile environment and supplied with sterile water and feed. The antimicrobial peptide NZ2114 was prepared by recombinant expression of *Pichia pastoris* X-33 with purity > 95% in Feed Research Institute, Chinese Academy of Agricultural Sciences (Zhang et al., 2014), the physicochemical properties of NZ2114 were shown in Table 1. All other chemical reagents used were of analytical grade.

**TABLE 1:** Physicochemical properties of NZ2114.

Peptide	Sequence	Purity	Length	MW (Da)	PI	Charge (+)	II
NZ2114	GFGCNGPW NEDDLRCH NHCKSIKG YKGGYCAK GGFVCKCY	>95%	40	4417.0	8.6	3	25.49

MW, molecular weight; PI, isoelectric point; II, instability index.

### Ability of NZ2114 against biofilms and bacteria of *Streptococcus dysgalactiae*

#### Minimum inhibitory concentrations determination

The MIC values of NZ2114 were measured by the microbroth dilution method (Wiegand et al., 2008). In brief, mid-log phase *S. dysgalactiae* CVCC 3938 cells were diluted to a suspension of 1 × 10^5^ CFU/mL with Tryptic Soy Broth medium (TSB, Qingdao Hope BioTechnology Co., Ltd.). NZ2114 was twofold diluted to final concentrations of 0.625–1280 μg/mL. The different concentrations of peptide (10 μL) and cell suspensions (90 μL) were added into a 96-well plate and incubated at 37°C for 16–18 h. The MIC value was defined as the lowest concentration at which there was no visible bacterial growth. All assays were performed in triplicate.

#### Biofilm growth kinetics measurement

*S. dysgalactiae* CVCC 3938 is a strong biofilm-producing strain according to the previous study (Zhang et al., 2021). The growth of biofilms was measured with a 3-(4,5-dimethylthiazole-2-yl)-2,5-diphenyl tetrazolium bromide (MTT) (Sigma) (Pires et al., 2011). In brief, mid-log phase *S. dysgalactiae* CVCC 3938 cells were diluted to a suspension of 1 × 10^8^ CFU/mL with TSB medium, 200 μL suspension and 100 μL MTT solution (0.5 mg/mL) were added into 96-well plates and incubated at 37°C for 72 h in the dark for further biofilm metabolism test. After incubation, MTT was displaced by the 150 μL DMSO dissolving solution and the optical density (OD) was determined at 570 nm using a microtiter plate reader (Cui et al., 2016).

#### Effect of NZ2114 to inhibit biofilm formation

The PBS suspension of mid-log phase *S. dysgalactiae* CVCC 3938 (1 × 10^8^ CFU/mL) was mixed with different final concentrations of NZ2114 or vancomycin (1–16 × MIC) and then incubated in 96-well plates at 37°C for 24 h. Untreated bacterial solution was the control group, and TSB was the blank control group. After incubation, the cells were washed with PBS for 3 times to remove floating cells and the effect of NZ2114 on early biofilm formation of *S. dysgalactiae* CVCC 3938 was assessed by the crystal violet staining. The inhibitory effect of NZ2114 on biofilms was determined according to the previous study (Yang et al., 2019).

#### Effect of NZ2114 to eradicate mature biofilm

Mid-log phase *S. dysgalactiae* CVCC 3938 was diluted to a suspension of 1 × 10^8^ CFU/mL with TSB medium and 200 μL bacterial suspension was incubated at 37°C for 24 h in 96-well plates. The supernatant was gently removed and 200 μL of NZ2114 or vancomycin at final concentrations of 1–16 × MIC were added and continued to incubate for 24 h. Untreated bacterial solution was the control group, and TSB was the blank control group. After incubation, the plates were washed with PBS for 3 times to remove the planktonic cells. The effect of NZ2114 on the eradication of mature biofilms of *S. dysgalactiae* CVCC 3938 was evaluated by crystal violet staining and the absorbance was measured as described above. All assays were performed in triplicate (Yang et al., 2019).

#### Effects of NZ2114 on bacteria in early biofilm

A volume of 200 μL mid-log phage *S. dysgalactiae* CVCC 3938 suspension (1 × 10^8^ CFU/mL in TSB medium) was added into 96-well plates and incubated at 37°C for 24 h. The plates were washed gently to remove the planktonic bacteria and incubated with NZ2114 or vancomycin at 37°C for 2 h. The untreated bacteria and free medium were used as control group and blank control group. After ultrasound, the attached bacteria were removed and colony count (Yang et al., 2019).

#### Bactericidal activity of NZ2114 against persister bacteria

In order to obtain persister bacteria from mature biofilms, a volume of 200 μL *S. dysgalactiae* CVCC 3938 bacterial suspension (1 × 10^8^ CFU/mL) was added into 96-well plates and incubated at 37°C for 24 h. After removed the planktonic bacteria, the plates were incubated with 100 × MIC vancomycin at 37°C for 24 h. After washed with PBS, the plates were treated with 16 × MIC NZ2114 or vancomycin at 37°C for 24 h for combatting the bacteria encapsulated in biofilm and then cultured in fresh PBS were used as a control. After ultrasound, the attached bacteria were removed and counted in colony forming units (Yang et al., 2019).

### Observation of biofilms by scanning electron microscope

According to the previous study, the biofilm of *S. dysgalactiae* CVCC 3938 was analyzed by SEM with some modifications. A volume of 360 μL *S. dysgalactiae* CVCC 3938 bacterial suspension (1 × 10^8^ CFU/mL) and 40 μL 160 × MIC NZ2114 or antibiotics were added into 24-well plates with sterile guide sheets and co-incubated at 37°C for 24 h. After incubation, the plates were washed 3 times with PBS to remove planktonic bacteria and the biofilm was fixed with 2.5% glutaraldehyde at 4°C for 24 h. The cells of biofilms were dehydrated with ethanol series (50, 70, 85, 95, and 100%) for three times with 15 min/time, dried by CO_2_, sputtered with platinum, and observed using a QUANTA200 SEM (FEI, Philips, Netherlands) (Zhang et al., 2021).

### Observation of biofilms by confocal laser scanning microscopy

To further study the inhibition and elimination effect of NZ2114 on biofilm and internal bacteria, biofilms were observed by CLSM. The sample pretreatment was consistent with SEM. After removed the planktonic bacteria, the biofilms were dyed with PI and SYTO9 (LIVE/DEAD BacLight Bacterial Viability Kit, ThermoFisher) for 15 min. Finally, the slides were washed with PBS and observed by Zeiss LSM880 confocal microscope (CLSM). The excitation/emission for these dyes are 480/500 nm for SYTO 9 stain and 490/635 nm for propidium iodide (Zhang et al., 2021).

### Efficacy of NZ2114 in murine mastitis model

#### *Streptococcus dysgalactiae* CVCC 3938-induced murine mastitis model

In order to study the therapeutic effect of NZ2114 against *S. dysgalactiae* CVCC 3938 on animals, a murine mastitis model was constructed. Specific pathogen free (SPF) ICR female mice at 4-day postpartum were selected. During the experiment, the mice were placed in an animal room with a temperature-controlled light-dark cycle (light: 8:00–20:00), supplied feed and water freely. The murine mastitis model was established as previous study with minor modifications (Li L. B. et al., 2017).

Briefly, the pups were separated from their lactating female mice 2 h before the experiment, and lactation was resumed 1 h before bacteria injection, so that the milk could be sucked up as much as possible. The mice were anesthetized with isoflurane through respiratory inhalation and placed them under the dissecting microscope, adjusting the focus so that the mouse nipples are clearly visible under the microscope. After disinfection with 75% alcohol, the nipple was gently fixed with sterile ophthalmic forceps in the left hand, and 100 μL bacterial suspension (1 × 10^5^ CFU/mL *S. dysgalactiae* CVCC 3938) or PBS was injected into the 4th pair of mammary glands through the milk ducts with a 100 μL microsyringe in the right hand.

### NZ2114 therapy in a murine mastitis model

In this study, a total of 16 lactating ICR mice were randomly divided into 4 groups: (1) Blank control group (Uninfected group); (2) Negative control group (Uninfected group); (3) NZ2114 treatment group; (4) Vancomycin treatment group. After challenge with *S. aureus* for 3 h, the dose of 100 μg/mammary gland NZ2114 and vancomycin was treated (dissolved in 100 μL PBS), and the blank control group was given the same volume of PBS. Mice were euthanized by cervical dislocation at 24 h, 3 day, 7 day after challenge, and the fourth pair of mammary glands were collected aseptically and weighed for bacterial counting.

### Inflammatory cytokine assay and histological evaluation of breast tissues

ELISA kits were used to determine IL-1β, IL-6, IL-10, TNF-α and MCP-1 indexes, and the remaining mammary tissues of each group were treated with 10% paraformaldehyde and stained with hematoxylin and eosin (HE) for histopathological analysis. All mouse experiments and animal welfare were performed in accordance with the protocol approved by the Animal Care and Use Committee-Ethical Committee for Experimental Animals (AEC-CAAS-20090609) of the Feed Research Institute, Chinese Academy of Agricultural Sciences.

### Statistical analysis

All data were analyzed by GraphPad Prism 8 (GraphPad Software, United States), and the results were given as means ± standard deviations (SDs). Statistical significance of groups was analyzed using the one-way ANOVA and Tukey multiple comparison.

## Results

### Ability of NZ2114 against biofilms and bacteria of *Streptococcus dysgalactiae*

#### Minimum inhibitory concentration determination

As shown in Table 2, NZ2114 displayed a potent antibacterial activity against *S. dysgalactiae*. The MICs of NZ2114 against *S. dysgalactiae* CVCC3938 was 0.11 μM, which was significantly superior to vancomycin (0.67 μM). For the clinical isolates *S. dysgalactiae* CAU-FRI 2,3, NZ2114 displayed a stronger antibacterial capacity with 0.23 μM than vancomycin (0.67 and 0.34 μM). Only for *S. dysgalactiae* CAU-FRI 1, NZ2114 showed an inferior activity (0.45 μM) compared with vancomycin (0.34 μM).

**TABLE 2:** The MIC values of NZ2114 against *S. dysgalactiae.*

Strain	NZ2114		Vancomycin	
		
	μ g/mL	μ mol/L	μ g/mL	μ mol/L
*S. dysgalactiae* CVCC 3938	0.5	0.11	1	0.67
*S. dysgalactiae* CAU-FRI 1	2	0.45	0.5	0.34
*S. dysgalactiae* CAU-FRI 2	1	0.23	1	0.67
*S. dysgalactiae* CAU-FRI 3	1	0.23	0.5	0.34

#### Biofilm growth kinetics measurement

**FIGURE 1:**
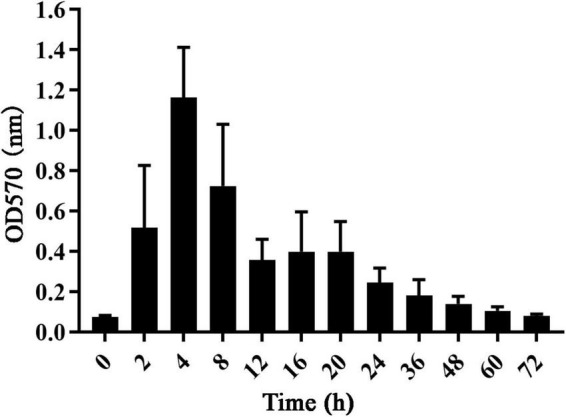
The growth curve of *S. dysgalactiae* biofilm.

The growth curve of *S. dysgalactiae* CVCC 3938 reflected the biofilm formation pattern. As shown in Figure 1, the absorbance value of *S. dysgalactiae* CVCC 3938 biofilm grew rapidly (OD_570_ 0.08–1.21) and the biofilm metabolism was vigorous in the first 4 h. At 12–20 h, the absorbance value tended to be stable (OD_570_ 0.36–0.40), and at 24–72 h, the absorbance values gradually decreased (OD_570_ 0.08–0.25).

#### Effects of NZ2114 to inhibit biofilm formation

**FIGURE 2:**
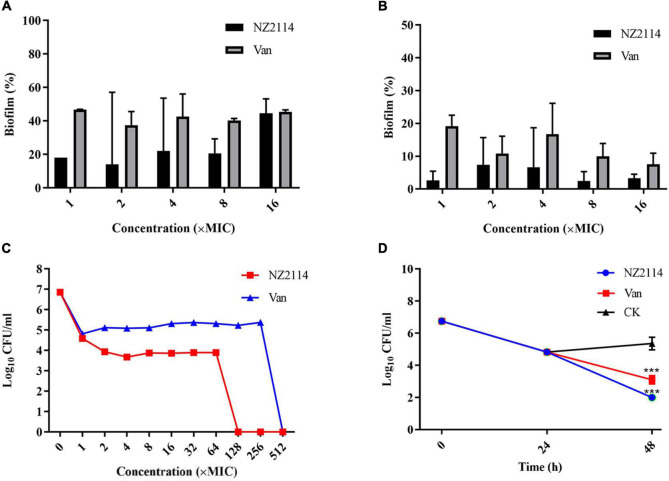
Effects of NZ2114 on *S. dysgalactiae* biofilms and bacteria in biofilms. **(A)** Inhibition effect of NZ2114 on biofilms formation. **(B)** Eradication effect of NZ2114 on mature biofilms. **(C)** Bactericidal activity of NZ2114 against the established biofilms. **(D)** Bactericidal activity of NZ2114 against persister.

The inhibitory effect of NZ2114 on the initial stage of biofilm formation was shown in Figure 2A, NZ2114 inhibited the initial biofilm of *S. dysgalactiae* CVCC 3938 by 77.9%–85.9% at concentrations of 1–8 × MIC, which was better than that of vancomycin (53.2–62.6%) at the same concentration. The results indicated that NZ2114 could effectively inhibit the initial biofilm formation of *S. dysgalactiae* at a certain concentration range.

#### Effects of NZ2114 to eradicate mature biofilm

As shown in Figure 2B, NZ2114 had a certain destructive effect on the mature biofilm, the biofilm eradication rates were 92.7–97.6% with increasing concentrations of NZ2114 at 2–16 × MIC, and the eradication rates did not change significantly when the concentration continued to increase, maintaining at about 96.7%, while the biofilm eradication rate of vancomycin-treated group was about 92.5% at the same concentration. Therefore, NZ2114 had the potential to eradicate mature biofilm.

#### Effects of NZ2114 on bacteria in early biofilm

As shown in Figure 2C, both NZ2114 and vancomycin reduced the number of bacteria encased in the biofilm. After treatment with 1–2 × MIC NZ2114, the colony number of *S. dysgalactiae* CVCC 3938 was rapidly decreased by 2.27–2.92 lg, but during 2–64 × MIC the number of colony remained stable, 128 × MIC NZ2114 completely killed the *S. dysgalactiae* CVCC 3938. The colony number of *S. dysgalactiae* CVCC 3938 was reduced by 2.04 lg after treated with 1 × MIC vancomycin, and until the concentration was 512 × MIC, vancomycin could completely eliminate the *S. dysgalactiae* CVCC 3938. Therefore, the killing efficiency of NZ2114 on bacteria in biofilm was significantly better than that of vancomycin.

#### Bactericidal activity of NZ2114 against persister

Through external application of antibiotics, bacteria were artificially induced biofilm to produce persister bacteria. A high dose of vancomycin (100 × MIC) was first added to a 96-well plate in which a mature biofilm of *S. dysgalactiae* CVCC 3938 had been formed. As shown in the Figure 2D, there still existed 6.8 × 10^4^ CFU/mL of persister bacteria encapsulated in the biofilm after treatment with 100 × MIC vancomycin for 24 h. After co-incubation with 16 × MIC NZ2114 for 24 h, the persister bacteria eradication rate of NZ2114 was 99.9%, significant difference with the untreated group (*P* < 0.001), while 16 × MIC vancomycin was less effective in inhibiting the persister bacteria. The number of viable bacteria in the biofilm with 16 × MIC vancomycin treatment decreased from 6.8 × 10^4^ to 1.4 × 10^3^ CFU/mL after 24 h, which was only one order of magnitude lower.

#### Biofilms observation by scanning electron microscopy

**FIGURE 3:**
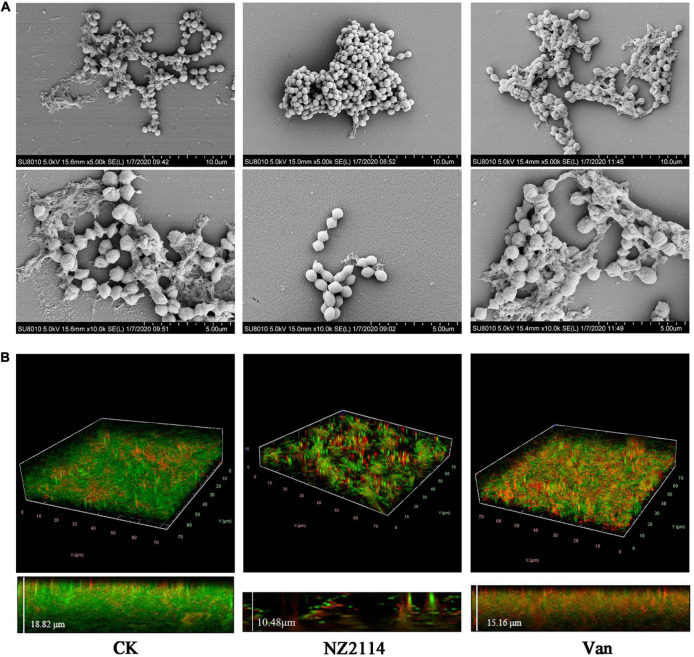
Effects of NZ2114 on *S. dysgalactiae* biofilms by SEM and CLSM observation. **(A)** Biofilms observation by SEM. **(B)** Biofilms observation by CLSM. Live cells are stained in green by SYTO9 and dead cells are stained in red by PI. CK: the untreated *S. dysgalactiae* biofilms. Van: vancomycin.

As shown in Figure 3A, the biofilm of the untreated control group was intact, and the whole biofilms were patchy and irregularly covered on the surface of the carrier, forming an irregular multi-layer structure. *S. dysgalactiae* CVCC 3938 cells were joined together by a sticky extracellular product secreted by the bacterium, which was encapsulated in the extracellular secretion. The vancomycin treatment group showed a decrease in the number of extracellular polymers and slight deformation of bacteria in the biofilm. After treatment with NZ2114, the biofilm became thin and loose, and the bacterial cells in the biofilm were deformed or ruptured.

#### Biofilms observation by confocal laser scanning microscopy

In order to further confirm the inhibition and eradication effect of NZ2114 on biofilm and internal bacteria, the treated *S. dysgalactiae* CVCC 3938 were observed by CLSM. As shown in the Figure 3B, the untreatment group formed biofilm of *S. dysgalactiae* CVCC 3938 with a thickness of 18.82 μm and more than 90% were live cells (stained with green color) in the field of view, after treatment with NZ2114, bacterial biofilm became significantly thinner (a thickness of 10.48 μm) and the number of dead bacteria increased (stained with red color), which was better than that of the vancomycin-treated group (a thickness of 15.16 μm). These results indicated that NZ2114 was superior to vancomycin in the inhibition and elimination of *S. dysgalactiae* CVCC 3938 biofilm and its internal bacteria.

### Efficacy of NZ2114 in murine mastitis model

#### *Streptococcus dysgalactiae* CVCC 3938-induced murine mastitis model

**FIGURE 4:**
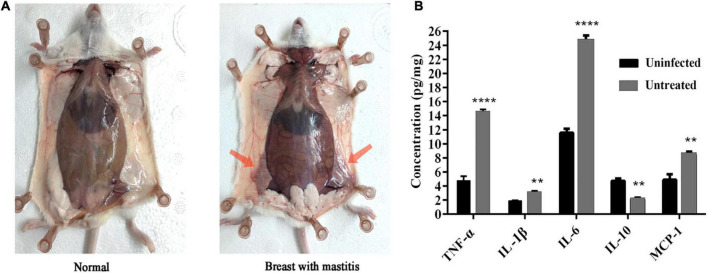
Establishment of murine mastitis model. **(A)** Pathological changes of mammary glands in mouse. **(B)** The change of inflammatory cytokine. A probability value of < 0.05 was considered significant. (*) Indicates the significance between uninfected and untreated groups. **p < 0.01; ****p < 0.0001. The results are given as the means ± SDs (*n* = 3).

Compared with the blank control group, the female mice infected with *S. dysgalactiae* CVCC 3938 for 24 h showed signs of swelling, erythema and hemorrhage with mammary tissue (Figure 4A). The acinar morphology was changed and acinar wall was thickened. In addition, levels of TNF-α, IL-1β, IL-6 and MCP-1 in mammary tissue were significantly increased (Figure 4B). The murine model in mice was successfully established.

#### NZ2114 therapy in a murine mastitis model

**FIGURE 5:**
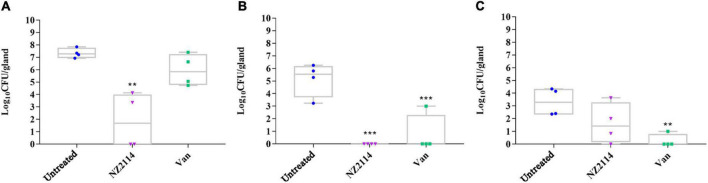
Effects of NZ2114 on bacterial loads in mammary tissue of *S. dysgalactiae* infected mice**. (A)** Treatment for 24 h; **(B)** treatment for 3 day; **(C)** treatment for 7 day. A probability value of < 0.05 was considered significant. (*) Indicates the significance between untreated and each of treatment groups. **p < 0.01; ***p < 0.001. The results are given as the means ± SDs (*n* = 3).

As shown in Figure 5, after challenge with *S. dysgalactiae* CVCC 3938 for 24 h, the bacterial number of untreated group was approximately 3.1 × 10^7^ CFU/gland. After treatment with NZ2114, the bacterial numbers in the mammary glands decreased significantly by 5.64 lg (from 7.33 to 1.87 lg, *P* < 0.01), 5.16 lg (from 5.16 to 0 lg, *P* < 0.01), and 1.69 lg (from 3.31 to 1.62 lg) at 1 d, 3 d and 7 d, respectively. For vancomycin treatment group, the bacterial load in the mammary glands decreased by 1.73 lg (from 7.33 to 5.96 lg), 2.16 lg (from 5.16 to 3 lg, *P* < 0.001), and 3.06 lg (from 3.31 to 0.25 lg, *P* < 0.01) at 1 d, 3 d and 7 d, respectively. Those results showed that the *in vivo* treatment effect of NZ2114 was better than that of vancomycin treatment group.

#### Inflammatory cytokine assay

**FIGURE 6:**
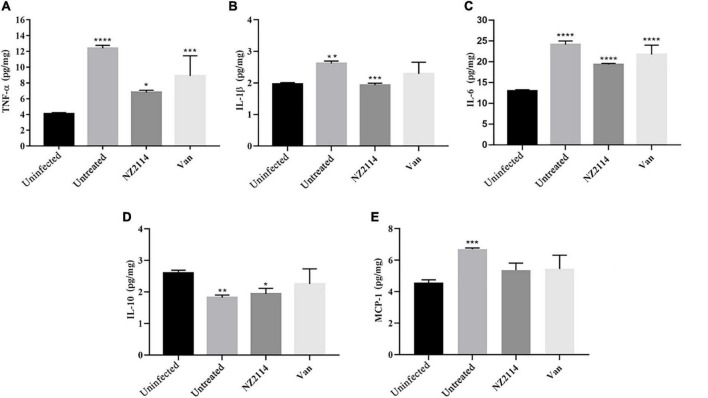
The protective effect of NZ2114 on the mammary gland of mice at the immune level. Mammary tissues were collected and the levels of **(A)** TNF-α, **(B)** IL-1β, **(C)** IL-6, **(D)** IL-10, and **(E)** MCP-1 were detected by using an ELISA kit after 24 h treatment, respectively. The analyses were measured by one-way ANOVA, with Duncan’s multiple comparisons test. All assays were performed in triplicate. The analyses were measured by one-way ANOVA, with Duncan’s multiple comparisons test. A probability value of <0.05 was considered significant. (*) Indicates the significance between control and each of treatment groups. **p < 0.01; ***p < 0.001; ****p < 0.0001. The results are given as the means ± SDs (*n* = 3).

To investigate the regulation effect of NZ2114 on immune factor of mammary gland in mice, the levels of inflammatory factors (TNF-α, IL-6 and IL-1β), anti-inflammatory factors (IL-10) and chemokines (MCP-1) in the mammary gland of mice were measured. As shown in Figures 6A–E, the levels of IL-1β, IL-6, IL-10, TNF-α and MCP-1 were 1.86–1.99, 19.05–19.57, 1.79–2.17, 6.48–7.13 and 4.35–5.88 pg/mg, respectively, after treatment with NZ2114. The levels of IL-1β, IL-6, TNF-α and MCP-1 in NZ2114 treatment group were significantly lower than those in negative control group and the regulatory effect of NZ2114 was comparable to those of the vancomycin treatment group.

#### Histological evaluation of breast tissues

**FIGURE 7:**
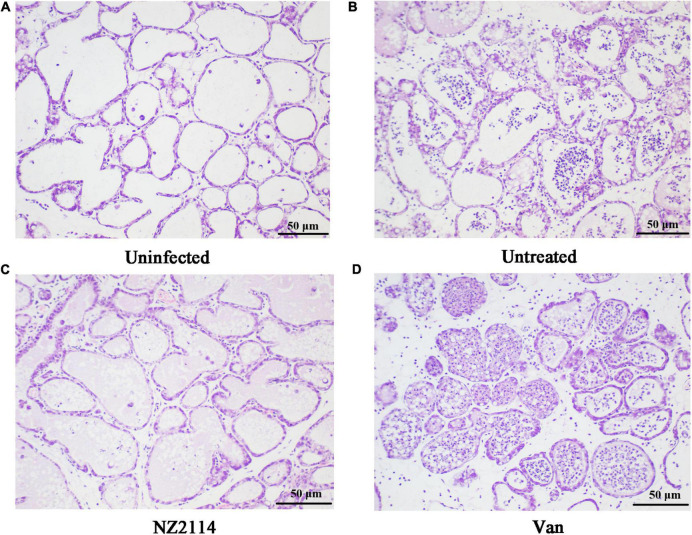
Histological evaluation of breast tissues. **(A)** Uninfected group; **(B)** Infected and untreated group; **(C)** NZ2114 treated group; **(D)** Vancomycin treated group.

As shown in Figures 7A–D, the inflammatory response of mammary tissue in NZ2114 and vancomycin treatment group were significantly improved, the infiltration of macrophages and neutrophils in the glandular follicles were significantly reduced, and the milk clot was reduced. Among them, NZ2114 treatment group was comparable to the normal level. The results indicated that NZ2114 had protective immune effects on mammary glands of mice.

## Discussion

Bovine mastitis is easy to relapse and difficult to cure, and *S. dysgalactiae* is one of the main pathogen. In this study, plectasin derived peptide NZ2114 displayed potent activity against *S. dysgalactiae* (Table 2). Therefore, the anti-biofilm and *in vivo* effects of NZ2114 on *S. dysgalactiae* were studied.

NZ2114 was demonstrated to inhibit the formation of early biofilm and eradicate mature biofilm of *S. dysgalactiae* CVCC 3938, which was significantly superior to vancomycin (Figures 2A,B). SEM and CLSM further demonstrated that NZ2114 could effectively reduce the biofilm thickness and bacterial colony of *S. dysgalactiae* CVCC 3938 (Figure 3). In addition, after treatment with antibiotics or attack by immune system of host, the bacteria could form small colonies of persister bacteria within the biofilm and remain in the host for a long time. Lewis et al., proposed that persister bacteria may play a role in biofilm tolerance and demonstrated that the presence of persister bacteria does prevent effective treatment of bacterial infections (Levin and Rozen, 2006). In this study, NZ2114 showed a potent role in the eradication of persister in early biofilm and mature biofilm, which was significantly better than that of vancomycin (Figures 2C,D). At the early stage of biofilm development, the adhesion ability of bacteria to the surface of natural polymer was increased under the action of cell wall associated adhesins. Therefore, the bacteria elimination effect during early biofilm showed that NZ2114 can inhibit the biofilm formation at the source. While for the mature biofilm, the elimination of biofilm plays an important role, then the persister bacteria in the biofilm could be dissociated, and the planktonic bacteria are further killed. NZ2114 not only had a more potent antimicrobial activity than its parent peptide plectasin *in vitro* and *in vivo* (Mygind et al., 2005; Andes et al., 2009; Brinch et al., 2009), but also more efficient bactericidal and biofilm removal effect than the other fungal defensin peptide P2 against *S. dysgalactiae* (Zhang et al., 2021), which may be related to the increased charge of NZ2114 (NZ2114 with 3 positive charges, plectasin and P2 with 1 positive charge). Recent studies have highlighted the potential use of AMPs to prevent biofilm formation or eradicate established biofilms (Rajasekaran et al., 2017; De Breij et al., 2018; Mwangi et al., 2019). However, there are also related researches of AMPs and compounds that only destroyed biofilms but did not kill bacteria. For example, De La Fuente et al. screened and obtained a strong anti-biofilm cationic nine-peptide 1037, which can inhibit the formation of biofilm at the concentration of 1/30 × MIC (>50% reduction in cell biomass). It disables the ability of bacteria to swim and aggregate and inhibit the expression of genes related to biofilm formation after interacting with bacteria, but the killing ability is relatively weak (de la Fuente-Núñez et al., 2012). Lee et al. had confirmed that extracts of various medicinal plants exhibited anti-biofilm activity against *S. aureus* without inhibiting the growth of cells in planktonic culture (Lee et al., 2013).

During lactation, the bacteria can easily pass through the milk ducts to the mammary gland. When a cow’s mammary gland is infected or damaged, the osmotic pressure balance between the cow’s milk and blood is disrupted, causing various cells and proteins in the blood to enter the milk pool or vesicles, which leads to changes in milk composition, κ-casein and lactoglobulin levels were reduced, whey protein, pH, and somatic cell count (SCC) were increased, respectively. This phenomenon significantly affects the technological quality of cows’ milk (Carroll et al., 1963; Pecka-Kiełb et al., 2016). What’s more, a large collection of white blood cells will block the ducts of the breast, resulting in the inability to discharge milk, a decrease in milk production, and even the cessation of lactation and loss of breast function. Because of the complex environment in the udder of dairy cows, *in vivo* testing is an essential part of evaluating new drugs for the treatment of mastitis. Several researchers have reported that the 4th pair of mouse mammary gland have been used as an infection model to test the therapeutic effects of gut microbiota, probiotics and phage (Wang et al., 2017; Hu et al., 2020; Ngassam-Tchamba et al., 2020). Therefore, a model of mastitis induced by *S. dysgalactiae* in mice was established in this work, which showed the typical symptoms of mastitis at the 4th pair of mouse mammary tissue as previously reported (Xi et al., 2020), the swelling, congestion and hemorrhage mammary tissue and the increased inflammatory cytokines. Compared with the vancomycin treatment group, NZ2114 displayed significant decrease of bacteria and immunomodulatory effects against *S. dysgalactiae*-induced mastitis, and the effect of NZ2114 was superior to that of vancomycin. However, the bacterial loads in mammary tissue were increased after treatment with NZ2114 for 7 days compared to treatment for 3 days, the reason may be related to the only once treatment after the challenge with *S. dysgalacetiae* within 7 days. Previous studies had proved that NZ2114 has more rapid bactericidal properties *in vitro* (Zhang et al., 2014) and intracellular (Wang et al., 2019) than vancomycin. Therefore, the treatment of mastitis *in vivo* with NZ2114 achieved a rapid bactericidal effect in the short term (1 d: from 7.33 to 1.87 lg, 3 d: from 7.33 lg to 0), but the concentration of NZ2114 in the breast was lower than the MIC value due to the absence of secondary administration, and the surviving *S. dysgalacetiae* was able to proliferate again. This result laterally proves that the antimicrobial peptide NZ2114 is not easily residual in the body, and the dosing interval needs to grope according to the *in vivo* pharmacokinetics. Vancomycin has the minimal colony count on day 7 *in vivo*, indicating that vancomycin is slow to take effect and has a long residual time in the body, and a 2–7 days milk abandonment period is needed after antibiotic treatment. NZ2114 could kill the two main pathogens *S. dysgalactiae* and *S. aureus* of mastitis, which is expected to be the best candidate for the treatment of bovine mastitis. And, it is reported that the industrial scale of 20 and 30 m^3^ special for AMPs fermentation and relative purification platform system were established in 2019 and 2021 in China for the first time, respectively (Gao and Wang, 2022), and a total amount of 49 Kg AMP products with a purity higher than 92% were harvested, which would be great helpful to evaluation and clinical trials on AMPs during drug development in next works.

*S. dysgalactiae* can not only form biofilm but also invade and survive within mammary epithelial cells, and persist for long periods, which can avoid the bactericidal action of antibiotics, resulting in persistent infection (Calvinho and Oliver, 1998). *Streptococcus* also can induce autophagy to increase inflammatory reactions of bovine mammary epithelial cells (Chen et al., 2022; Qi et al., 2022). Because of the low intracellular accumulation and special intracellular environmental effects, it is difficult for the antibiotics to play a good bactericidal effect in intracellular. In this study, NZ2114 not only killed bacteria *in vitro*, but also completely eliminated *S. dysgalactiae* in mammary gland tissue, which may be related to the intracellular bactericidal efficacy of NZ2114. The previous studies have shown that NZ2114 was internalized in the bovine mammary epithelial cells *via* clathrin-mediated endocytosis and micropinocytosis, and maintained potent intracellular antibacterial activities (Wang et al., 2018). NZ2114 has dual effects of anti-biofilm and anti-intracellular bacteria, which may explain the main reason that NZ2114 could effectively cure murine mastitis.

In general, *in vitro* and *in vivo* antibacterial and antibiofilm effects of NZ2114 on *S. dysgalactiae* CVCC 3938 were systemically studied for the first time. NZ2114 revealed the inhibition/eradication effect on the early/mature biofilm of *S. dysgalactiae* and vancomycin-resistant persister encapsulated the biofilm. Furthermore, NZ2114 is an excellent drug for the treatment of *S. dysgalactiae*-induced mastitis. These results indicated NZ2114 is a potential effective drug for clinical treatment of *S. dysgalactiae* infection.

## Data availability statement

The original contributions presented in this study are included in the article/supplementary material, further inquiries can be directed to the corresponding authors.

## Ethics statement

All animal procedures used in this study were approved by the Animal Care and Use Committee of Feed Research Institute, Chinese Academy of Agricultural Sciences (Permit Number: 20150309) and were performed in accordance with ARRIVE (Animals in Research: Reporting *in vivo* Experiments) guidelines.

## Author contributions

QZ, NY, RM, DT, HF, and JW conceived and designed experiments. NY and QZ carried out all the experiments. NY, QZ, DT, and JW contributed in writing. JW and HF contributed in funding acquisition. YH and XM contributed to the materials and reagents. RM contributed in modifying figures. All authors contributed to the article and approved the submitted version.
